# A Systematic Review of the Robson Classification for Caesarean Section: What Works, Doesn't Work and How to Improve It

**DOI:** 10.1371/journal.pone.0097769

**Published:** 2014-06-03

**Authors:** Ana Pilar Betrán, Nadia Vindevoghel, Joao Paulo Souza, A. Metin Gülmezoglu, Maria Regina Torloni

**Affiliations:** 1 UNDP/UNFPA/UNICEF/WHO/World Bank Special Programme of Research, Development and Research Training in Human Reproduction, Department of Reproductive Health and Research, World Health Organization, Geneva, Switzerland; 2 Maternal Child Clinic, Calgary, Canada; 3 Department of Social Medicine, Ribeirao Preto Medical School, University of São Paulo, Ribeirao Preto, SP, Brazil; 4 Brazilian Cochrane Centre, São Paulo, Brazil, and Department of Internal Medicine, São Paulo Federal University, São Paulo, Brazil; University of Aberdeen, United States of America

## Abstract

**Background:**

Caesarean sections (CS) rates continue to increase worldwide without a clear understanding of the main drivers and consequences. The lack of a standardized internationally-accepted classification system to monitor and compare CS rates is one of the barriers to a better understanding of this trend. The Robson's 10-group classification is based on simple obstetrical parameters (parity, previous CS, gestational age, onset of labour, fetal presentation and number of fetuses) and does not involve the indication for CS. This classification has become very popular over the last years in many countries. We conducted a systematic review to synthesize the experience of users on the implementation of this classification and proposed adaptations.

**Methods:**

Four electronic databases were searched. A three-step thematic synthesis approach and a qualitative metasummary method were used.

**Results:**

232 unique reports were identified, 97 were selected for full-text evaluation and 73 were included. These publications reported on the use of Robson's classification in over 33 million women from 31 countries. According to users, the main strengths of the classification are its simplicity, robustness, reliability and flexibility. However, missing data, misclassification of women and lack of definition or consensus on core variables of the classification are challenges. To improve the classification for local use and to decrease heterogeneity within groups, several subdivisions in each of the 10 groups have been proposed. Group 5 (women with previous CS) received the largest number of suggestions.

**Conclusions:**

The use of the Robson classification is increasing rapidly and spontaneously worldwide. Despite some limitations, this classification is easy to implement and interpret. Several suggested modifications could be useful to help facilities and countries as they work towards its implementation.

## Background

In 1985, The World Health Organization (WHO) stated: “There is no justification for any region to have a caesarean section (CS) rate higher than 10–15%” [1]. Despite the lack of scientific evidence indicating any substantial maternal and perinatal benefits from increasing CS rates, and some studies showing that higher rates could be linked to negative consequences in maternal and child health [2]–[4], CS rates continue to increase worldwide, particularly in middle- and high-income countries, and have become a major and controversial public health concern [5], [6].

The lack of a standardized internationally-accepted classification system to monitor and compare CS rates in a consistent and action-oriented manner is one of the factors preventing a better understanding of this trend and underlying causes [7]. In 2011, a systematic review and critical appraisal of available classifications for CS concluded that women-based classifications in general, and Robson's 10-group classification in particular, would be in the best position to fulfill current international and local needs [8]. The review recommended that efforts to develop an internationally applicable classification should be most appropriately placed in building upon this classification. Robson proposes a system that classifies women into 10 groups based on their obstetric characteristics (parity, previous CS, gestational age, onset of labour, fetal presentation and number of fetuses) without needing the indication for CS [7]. Table 1 shows the definitions of each group. Since this system can be applied prospectively, and its categories are totally inclusive and mutually exclusive, every woman who is admitted for delivery can be immediately classified based on these few basic characteristics which are usually routinely collected by obstetric care providers worldwide. If used on a continuous basis, some studies suggest that this classification system can provide critical assessment of care at delivery and be used to change practice [7], [9].

**Table 1:** Obstetric characteristics of women included in each of the 10 groups of the classification; subdivisions proposed by the authors of the 73 included studies, and the number of studies proposing each subdivision by group of Robson.

Group	Women included					
		Augmentation vs no augmentation	Spontaneous/induced/CS before labour	With/without previous uterine scar	Previous vs no previous VD	One previous scar vs >1 previous scar
1	Nulliparous with single cephalic pregnancy, ≥37 weeks gestation in spontaneous labour	1				
2*	Nulliparous with single cephalic pregnancy, ≥37 weeks gestation who either had labour induced or were delivered by CS before labour					
3	Multiparous without a previous uterine scar, with single cephalic pregnancy, ≥37 weeks gestation in spontaneous labour					
4*	Multiparous without a previous uterine scar, with single cephalic pregnancy, ≥37 weeks gestation who either had labour induced or were delivered by CS before labour					
5	All multiparous with at least one previous uterine scar, with single cephalic pregnancy, ≥37 weeks gestation		8#		6	6
6	All nulliparous women with a single breech pregnancy		1			
7	All multiparous women with a single breech pregnancy including women with previous uterine scars		1	2		
8	All women with multiple pregnancies including women with previous uterine scars		4	3		
9	All women with a single pregnancy with a transverse or oblique lie, including women with previous uterine scars		2	2		
10	All women with a single cephalic pregnancy <37 weeks gestation, including women with previous scars		3	2		

*Often divided into 2a and 4a (inductions) and 2b and 4b (pre-labour CS): These were originally proposed by Robson in 2001 and have been used/proposed by 27 articles.

# This includes one article that proposed Trial of labour after CS (TOLAC) *vs* No TOLAC.

Since 2001, when the Robson classification (also called the 10-group classification) was proposed, many facilities and countries have incorporated it in their routine clinical practice as a tool to monitor CS rates in their population and to evaluate the impact of changes in management that may alter these rates [10]–[14]. However, to the best of our knowledge, there is no systematic synthesis and assessment of the experiences, opinions and challenges encountered by users in their facility or country. This information could help units as they work towards the implementation of the classification to plan the necessary steps on more realistic grounds, to be aware of the most challenging issues, and to address critical potential pitfalls in their setting.

Against this background, we set out to conduct a systematic review of the literature to gather the experience of users related to the pros and cons of the adoption, implementation and interpretation of the Robson classification, as well as their adaptations, modifications or recommendations on the use of this classification.

## Methods

This systematic review was conducted following a protocol specifically designed for this purpose and reported according to the recommendations of the PRISMA statement [15] and the Meta-analysis Of Observational Studies in Epidemiology group (MOOSE) [16].

### Type of study designs

Any study that described the experience of using the Robson classification was eligible for inclusion regardless of the objective and design of the study or the context or setting (e.g. nationwide, facility-based) in which it was applied.

### Type of participants

Any study presenting the use of the Robson classification in any group of women was eligible for inclusion regardless of the women's obstetric or medical characteristics, level of risk, education or socio-economic status.

### Type of implementation of the Robson classification

We included studies presenting the use of the Robson classification involving any number of patients, for any period of time, for any reason (e.g. audit and feedback, monitor trends, document effectiveness of interventions), to assess any outcome (e.g. rates of CS, maternal or perinatal indicators, patient satisfaction, costs). Studies that used variations of the Robson classification (e.g. analyzing only Robson groups 1 and 2 instead of the 10 groups, or splitting or lumping groups) were eligible for inclusion as long as they described the changes in sufficient detail to be replicable.

### Exclusion criteria

We excluded studies that were strictly theoretical or described opinions that were not based on actual experiences of the authors related to the use of the classification or if the definitions used to categorize women in the groups were dubious or unclear. There were no language or country restrictions in this review.

### Search strategy for the identification of studies

The search strategy was developed with the assistance of a librarian experienced in electronic search strategies for systematic reviews, from the Brazilian Cochrane Center. Four electronic databases were searched: Medline, Embase, CINAHL and LILACS from January 2000 to 18 January 2013 (see complete search strategy in File S1).

The references of all articles selected for full-text evaluation were also checked for additional potentially relevant studies not identified through the electronic search. Authors were contacted through e-mail for additional data, when necessary. Dr Michael Robson, creator of the classification was contacted to inquire about unpublished material from units that had implemented the classification.

### Screening, data extraction template

All citations identified from the electronic searches were downloaded into Reference Manager software version 11 and duplicates were deleted. Two investigator (APB, MRT) independently screened the title and abstract to select potentially relevant citations for full-text reading. All selected articles were independently read by two reviewers (APB, MRT) and those fulfilling the aforementioned selection criteria were included in the review. Disagreements in the process of screening and selection of articles were discussed until consensus was reached. In cases of studies with more than one publication, the latest and/or more complete version was used. Data extraction was performed by two reviewers (APB, MRT; independently and in duplicate) using a standardized data-extraction template specially designed for this review. The information was extracted and discussed until full agreement. A final extraction form was filed for each study.

Information captured for each article included: 1) objectives of the study; 2) country, year, setting, type of institution, time period when the classification was used, number of women/deliveries included, completeness, source of data and average CS rate; 3) observations, comments or criticisms to the overall classification or to any of the 10 groups, adaptations or suggestions proposed to improve the classification, facilitators and barriers identified for its use and implementation; and 4) definitions of the variables used in the construction of the groups of the classification.

### Data extraction and synthesis

A thematic synthesis approach [17] and a qualitative metasummary method [18] were used. We also followed the principles of the Cochrane Qualitative Research Methods Group [19]. In brief, we followed three steps to systematically extract and synthesize the views from the authors in the original articles: (a) line-by-line coding to extract the key concepts, usually presented in the Results, Discussion or Methods section; (b) organization of these key concepts to construct “descriptive” themes/topics that formed the skeleton of the structure of the analysis; and (c) development of analytical themes based on the synthesis of the experiences and recommendations of authors of the original articles. This process was performed manually, i.e. without the use of a specific software. The detailed description is depicted in File S2. Three investigators (APB, MRT, NV) coded the concepts, developed the descriptive themes and then the analytical themes, with regular discussions and meetings until reaching full agreement. To assess the relative magnitude of each abstracted concepts, we calculated their frequency effect size [18]. For each concept, the effect size was calculated by dividing the number of reports containing the concept (minus any report derived from the same study and therefore representing a duplicate) by the total number of reports (minus any report derived from the same study and therefore representing a duplicate). In our review, there were no duplicate reports.

## Results

**Figure 1:**
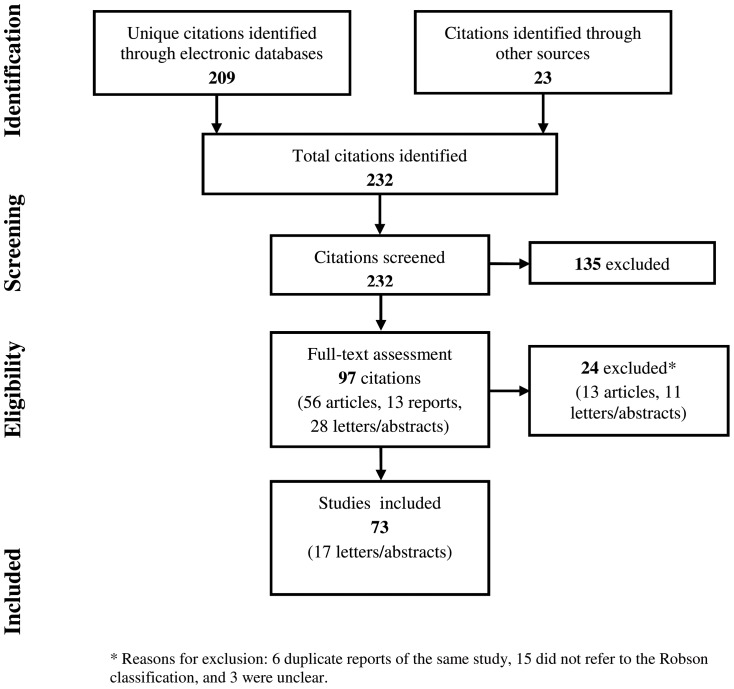
Flowchart of the systematic review.

The electronic search strategy yielded 273 citations that were reduced to 209 after removing 64 duplicates. An additional 23 records were identified through other sources. After screening titles and abstracts, 97 citations were selected for full-text assessment and 73 were included in this review (see flowchart in Fig 1).

**Figure 2:**
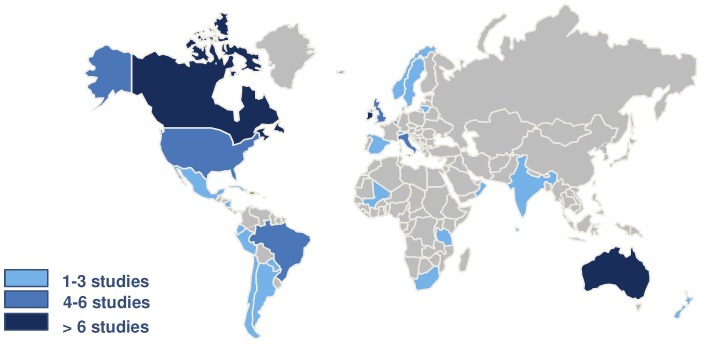
Distribution of the 73 articles on Robson's classification according to country of origin.

Table 2 presents the main characteristics of the 73 included studies, which report on the use of the Robson classification in over 33 million women. Two thirds of the included studies were published in 2010 or after and presented data collected (either retrospectively or prospectively) from 1974 to 2012. The overall CS rate in the 63 articles that reported this figure ranged from 5% (1974) [20] to 53.5% (2010) [21]. Most of the studies were either cross-sectionals (40%) or trend analysis (36%) using the 10 groups over time. Figure 2 shows the geographical distribution of the 73 studies included in this review; almost 70% of them were conducted in developed regions (Europe, North America and Oceania). Over 70% of the studies reported on the use of the classification at hospital-level and hospital records were the main source of data (Table 2).

**Table 2:** Characteristics of 73 studies that reported the use of Robson's classification.

Characteristics	N (%)
**Type of manuscript**	
Articles in peer-reviewed journals	42 (57.5)
Congress Abstracts	14 (19.2)
Reports	13 (17.8)
Other*	4 (5.5)
**Type of study**	
Cross-sectional group analysis	32 (43.8)
Trend analysis	26 (35.6)
Before-and-after intervention	6 (8.2)
Advocacy/Guidelines study	6 (8.2)
Other (letter, commentary, etc)	3 (4.1)
**Region**	
Europe	26 (35.6)
North America	14 (19.2)
Oceania	9 (12.3)
South America	8 (11.0)
Asia	5 (6.8)
Africa	4 (5.5)
Multi-country**	2 (2.7)
Not applicable***	5 (6.8)
**Country Income Group** #	
High Income	58 (69.9)
Upper Middle Income	17 (20.5)
Lower Middle Income	5 (6.0)
Low Income	3 (3.6)
**Setting**	
Hospital based	53 (72.6)
Tertiary hospital	28 (52.8)
Level not stated	11 (20.8)
Multiple hospitals	14 (26.4)
Population-based	14 (19.2)
Not applicable	4 (5.5)
Not specified/Unclear	2 (2.7)
**Source of data**	
Hospital records	40 (54.7)
Birth certificate/registry	12 (16.4)
Perinatal database	12 (16.4)
Not applicable	4 (5.5)
Not specified/Unclear	5 (6.8)
**Number of women classified per study**	
>50,000	12 (16.4)
10,000–50,000	23 (31.5)
<10,000	31 (42.4)
Not applicable	4 (5.5)
Not specified/Unclear	3 (4.1)
**Coverage of the classification** ♦	
≥95% of all delivered women	14 (19.2)
<95% of all delivered women	4 (5.5)
Not applicable	4 (5.5)
Not specified/Unclear	51 (69.9)

*3 letters, 1 unpublished manuscript

**1 study with 8 South American countries, 1 study with 9 countries including Oceania, North America and Europe

***Commentaries and letters

# World Bank Income Group Classification http://data.worldbank.org/about/country-classifications/country-and-lending-groups#Low_income. Out of 83 as some studies had multiple countries.

**^♦^**Coverage is defined as the number of women included in the classification as a percentage of the total number of women delivered during the study period.

In line with the thematic synthesis approach [17], the findings of this review are presented under three descriptive themes: design/purpose of the classification, implementation of the classification, and interpretation of the information arising from the classification. *Design/purpose* includes issues related to the principles, notion, idea, structure, and construct of categories or groups of the classification and its purpose or function. *Implementation* refers to mechanisms and processes related to how the classification is put into use, including how the required information is obtained, who collects this information, definitions of the variables used, quality assurance, and other elements like the use of software versus manual notation. *Interpretation* refers to issues relevant for the understanding of the information and data that emerges from the classification and its implementation. Table 3 shows the pros and cons of the Robson classification under each of these three themes and the percentage of studies that mentions each concept. The paragraphs below present the most recurrent concepts.

**Table 3:** Pros and cons of the Robson classification as experienced and reported by the authors and users in 73 articles included in this systematic review, and effect size (the proportion of articles containing each concept).

Pros as experienced by users/authors	Effect size (%)	Cons as experienced by users/authors	Effect size (%)
**Design/purpose of the Robson classification**			
Robust, simple, reproducible informative and useful tool for comparisons, on-going surveillance and audit [7], [9]–[14], [21], [24], [26], [28], [30], [33]–[37], [43], [46], [47],	48	Identifies contributors to CS rate but not the reasons for performing a CS (indications) or explanations for differences [7], [9], [13], [31], [36], [46], [56], [60], [78], [80]	14
Allows studying rates in more homogeneous groups of women in whom to focus interventions (e.g. management guidelines) and audits/monitoring [10], [12], [13], [28], [30], [33], [37], [42], [52], [63], [72], [74], [76]	18	Some heterogeneity remains within groups as some important variables that influence the rate of CS are not included in the classification, such as: pre-existing clinical conditions, obstetric complications, indications and methods for induction, exact gestational age and subgroups of preterm birth, maternal age and BMI [14], [20], [56], [78], [81]	7
Can be used as an intervention to reduce CS [22]–[24]	4	The classification is unable to directly evaluate the relationship between CS and outcomes [60], [78]	3
Useful for both public health and clinical settings [12], [56]	3	For inter-hospital comparisons, other statistical methods (e.g. adjusting) are necessary to account for maternal and fetal factors not included in the classification [76]	1
Offers flexibility for local adaptation [10]	1		
Allows analysis of the contribution of induction to the overall CS rate [9]	1		
Some components of the classification allow for data validation (self-validation of the classification) [9]	1		
**Implementation of the classification**			
Variables are readily available and well defined which minimizes inconsistencies [11]–[13], [25], [37], [57], [79]	10	Although minimal resources are necessary to implement the classification, the very limited resources available for systematic CS audits in some settings is one factor that prevents more use of the classification (and any audit) [33]	1
Not requiring indications is an advantage as indications are insufficiently registered and potentially subjective [7], [10], [25]–[27]	7		
Easily implemented across a range of countries, hospitals and systems (including low-resource settings) [32], [33], [82]	4		
Requires minimal resources [25], [71]	3		
Raises staff awareness about data; its use may results in improvements in quality of data collection and documentation in general [25],[28]	3		
It does not require sophisticated software [7]	1		
Raises staff awareness of CS rates; staff welcomes this information [28]	1		
**Interpretation of results**			
Value lies in its prospective use with continuous feedback to the staff, allowing targeting specific groups of women to improve care, monitor effectiveness of implemented strategies and ultimately, improve outcomes [12], [26], [41], [42], [65]	7	Inter-hospital comparisons have a great potential, however, when adjustments are incorporated, the likely inconsistencies in coding discharge may challenge accuracy of assessment of outcome and risk factors [76]	1
Potential as a benchmarking tool which enables international comparisons without major interpretation difficulties [12], [13], [28], [79]	6		
Leads to additional analyses that may not have been made by traditional observation of CS rates [9], [31], [78]	4		
Challenges some common myths about causes of increasing CS rates [28]–[30]	4		
Demonstrates that the overall CS rate is affected by both the magnitude of the CS rate and the relative size of each group [58]	1		

### Pros of the Robson classification as experienced by users

Users praise the simplicity, robustness, reproducibility and flexibility of the classification; and the fact that the classification is clinically relevant and categorizes women prospectively which in turn allows the implementation and evaluation of interventions targeted at specific groups. The classification itself can be used as an intervention to reduce CS rates [22]–[24] and help to analyze the contribution of inductions to the overall CS rate [9]. An inherent advantage of the classification is that it allows self-validation since some groups can act as controls. For instance, group 9 (women with a fetus in a transverse or oblique lie) is expected to represent less than 1% of all women admitted for delivery and to have a CS rate of close to 100%. Numbers that differ significantly from these values indicate the possibility of problems with data collection [9].

The resources, software and variables needed to implement the classification are considered minimal, making it suitable for low-resource settings. In addition, “not requiring indications for CS” is an advantage [7], [10], [25]–[27] because of the variability and potential subjectivity when using indications to classify CS, and because these are insufficiently registered in some settings. This classification challenges traditional myths about alleged drivers of increasing CS rates, such as breeches or multiple pregnancies [28]–[30].

### Cons of the Robson classification as experienced by users

Users report that the *basic* Robson classification identifies the contributors to the CS rate but does not provide insight into the reasons (indications) or explanations for the differences observed. The classification does not take into account other maternal and fetal factors that significantly influence the rate of CS (e.g. maternal age, pre-existing conditions such as BMI or complications) and therefore additional statistical methods (e.g. adjusting) are necessary to account for these factors.

### Recommendations by users

Table 4 shows the modifications, adaptations or recommendations suggested by the users of the classification and the percentage of studies that mentions each recommendation. The paragraphs below present the most recurrent modifications.

**Table 4:** Modifications, adaptations and recommendations for implementing and interpreting the Robson classification according to the authors/users of the 73 articles included in this systematic review, and effect size (the proportion of articles which recommended each of them).

Recommendations by users/authors	Effect size (%)
**Design/purpose of the Robson classification**	
Additional subcategories of the 10 groups is recommended/used to further decrease heterogeneity of each group (see Fig 3 for sub-groups proposals) [7], [9], [12], [14], [20], [23], [27], [29]–[31], [48], [53], [54], [57], [58], [60]–[68], [80], [83]	36
Within group analysis for site- and population-specific relevant variables	
• Indications and maternal morbidities can be analyzed efficiently by group; indication should be recorded in a hierarchical standardized manner [7], [9], [11], [12], [22], [29], [31], [44], [60], [63], [68], [78], [81]	18
• Indication for inductions can be analyzed in the relevant groups in a standardized manner allowing for analysis of contribution of inductions to the overall CS rate [31], [63], [68]	4
• Use of operative vaginal delivery can be analyzed in the relevant groups. Analysis not only of CS rates but also of the rate of spontaneous vaginal delivery (non-operative deliveries) is an important concept because of the inverse relationship between operational deliveries and CS [40], [47]	3
• In addition, other variables, aspects and characteristics of women can be analyzed within each group: gestational age, body mass index (BMI), age, medical conditions, fetal distress, race, staff shifts, etc. [9], [12], [13], [20], [33], [39], [45], [46], [62], [64], [68], [76]–[78], [81], [84]	22
Merging Group 1 and Group 2 to gather all nulliparous may be useful for certain analysis. Other merges are possible and have been proposed (e.g. merge of groups 6 through 10, groups 1 and 3, groups 2 and 4) [9], [21], [32], [33], [39], [49], [50], [81]	11
A group “99” can be created for women who cannot be classified (e.g. women with missing information) [13], [56], [57]	4
Maternal satisfaction with the experience of the delivery should also be collected [31]	1
**Implementation of the classification**	
Regular audits for continued data quality improvement should be in place as quality of data is, in general, challenging [74]	1
There is lack of consensus or proposed definitions for variables/concepts that are critical for the classification (See Table 5 for definitions): [56]	1
• Definitions need to be clear and stated: e.g. vertex vs. cephalic, induction vs. augmentation [31], [63]	3
• A common agreement on when to diagnose the start of labour is needed, particularly in case of premature rupture of membranes (PROM) [47]	1
For accuracy and validity, efforts to avert incomplete and missing information need to be in place: [13], [58]	3
• Difficulties in availability of the exact fetal presentation have led some users to categorize women who belonged in Group 9 (transverse and oblique lie) into Groups 6 and 7 as breeches [59]	1
• Accurate assignment of gestational age may be challenging in certain settings [25]	1
• When multiple sources are used (e.g. population-based national level studies), depending on the source of the data (e.g. birth certificates), not all the variables are available (e.g. CS before labour, transverse/oblique lie) and correlation between data in birth certificates and medical records is not guaranteed [52], [70], [81]	4
• If the variable “induced” is not easily available, it would not be possible to present groups 1, 2, 3 and 4 separately [27]	1
• Training helps to ensure that no data is missing and all women are correctly classified. Educational effort are needed especially for classifying fetal presentation and position (e.g. difference between occiput transverse presentation and transverse lie) [32], [56], [65]	4
Although repeatedly proposed, collecting additional information (e.g. indication, maternal characteristics, etc) may pose a challenge due to poor quality of maternity data and non-standardized definitions; particular efforts need to be put in place to maintain quality of data [26]	1
Involve, engage and develop ownership; a collaborative effort by clinicians, midwifes, nurses and data management personnel will achieve more complete and accurate recording on the patient record, and timely data collection to ensure high quality information [63]	1
**Interpretation of results**	
Understanding how to interpret the data is critical for clinicians in the context of everyday clinical practice [78]	1
Using the classification, the optimal CS rate should be calculated after analysis of outcomes for each group [9], [45], [52]	4
Allows to assess and monitor effectiveness of implemented interventions [42], [65]	3
Novel uses such as subgroup assessment have been proposed (e.g. women with diabetes and women with systemic lupus erythematosus); or examining outcomes other than CS (e.g. peripartum hysterectomy) as part of a new system to monitor patient safety [26], [51], [69], [75]	6

Ten groups constitute the backbone of the Robson classification. However, many authors used or proposed further subclassifications in each group or merging of groups. Among the 58 studies presenting data using the classification, 34 gave data using the original 10 Robson groups with no subgrouping [9]–[12], [14], [25]–[28], [31]–[55], 18 studies presented their data using subgroups or adding new groups [13], [20], [23], [29], [30], [56]–[68] and seven studies used less than 10 groups either by focusing on only one or two groups or by combining groups [21], [22], [59], [69]–[72]. One study proposed both merging and splitting of categories [59].

Table 1 shows the number of studies proposing each subdivision for each Robson group. All but one proposed the subdivision originally suggested by Robson for Groups 2 and 4 into induced (2a and 4a) and CS before labour (2b and 4b). The two most popular subdivisions (useful in several Robson groups) were (i) spontaneous labor/induced labour/CS before labor (Groups 5 through 10), and (ii) without previous uterine scar/with previous uterine scar (Groups 7 through 10). Several different subdivision were proposed for Group 5. A detailed list of the articles suggesting each subdivision is provided in File S2.

Merging Robson groups for specific analysis was also proposed. Most frequent were merging groups 1 and 2 to analyze all nulliparous women together [9], [32], [33], [39], [49] or all multiparous women by merging groups 3 and 4 [9]. Users also suggested collecting additional variables (such as indications for induction and CS or epidemiological and demographic variables) for within group analyses (Table 4). For example, indications for CS could be used within each group and in a hierarchical and standardized manner using the Anderson model [73].

Because ensuring continued quality data collection can be challenging, users recommended regular audits [74]. In particular, users reported challenges in extracting data on fetal presentation and position, induction vs. augmentation, and gestational age; they emphasized the need for training, in both developed and developing countries (see Table 4). In addition, although the collection of additional variables was repeatedly proposed, users warned that the collection of these variables (e.g. indication, reasons for induction, obesity, age) may pose challenges due to poor quality of data and non-standardized definitions. Engaging and involving staff may result in more complete and accurate recording on the patient record, timely collection and better quality data [25], [28].

#### Definitions of core variables in the Robson classification

Although the 10 groups of the Robson classification are constructed by using a few basic core variables collected from every woman admitted for delivery, there was some variation in the definitions of these parameters, as shown in Table 5. While no article presented a definition of spontaneous labour, four defined induced labour [20], [30], [32], [39]. Multiple definitions were used for what is considered a “birth” and therefore which pregnant women can be included in the classification [13], [21], [23], [27], [33], [41], [45], [54], [59] (see Table 5).

**Table 5:** Definitions proposed by users for variables required in the Robson classification.

Variable	Definitions suggested by users
**Parity**	Nulliparous: para 0 irrespective of gravidity [20], [81]
**Spontaneous labour**	No definitions mentioned
**Induced labour**	• Use of any medication or amniotomy when not in labour, rather than accelerate labor, that had already commenced spontaneously [20], [39], [81]
	• Only pharmacological induction [30]
**CS before labour**	No articles defined CS before labour.
	Elective/emergency as a way to define a CS performed in a women before labour or a woman who is already in labour [33], [65]
**Lie**	No definitions mentioned
**Presentation**	Vertex as a proxy for cephalic [56]
**Term Birth**	• Birth occurring at or after 37 weeks [20], [38], [54], [81]
	• >2500 g as a proxy [25]
**Singleton**	No evidence of multiple gestation after the 1st trimester [20], [81]
**Birth (live birth/stillborn GA or birth weight)**	• Live birth and Stillbirths Gestational age ≥20 weeks [13]
	• Gestational age ≥23 weeks [59]
	• Birthweight >500 g [23]
	• Live births with birthweight >500 g [41]
	• Gestational age ≥20 weeks or birthweight >400 g [27]
	• Live birth and stillbirths gestational age ≥20 weeks and birthweight >400 g [54]
	• Gestational age ≥22 weeks or birthweight >500 g [21], [33]
	• Live births gestational age ≥22 weeks and birthweight >500 g [45]

Understanding how to interpret the data from the classification is considered critical for the clinicians. From the public health perspective, users suggest that the optimal CS rate should be calculated after analysis of outcomes in each Robson group. Some novel uses of the classification have been proposed (See Table 4) [26], [51], [69], [75].

## Discussion

This review identified 73 manuscripts presenting the experiences of users on the pros and cons of the adoption, implementation and interpretation of the Robson classification for CS. Our findings show that, despite the lack of official endorsement by any international institutions or any formal guidelines, the use of the Robson classification is increasing rapidly and spontaneously worldwide. In this scenario, the experience and views of the users are a rich source of knowledge and guidance.

According to the users, the main strengths of the Robson classification are the simplicity of its design, the validity of its purpose, its ease of implementation and directness of initial interpretation. This classification has the capacity to overcome the main drawbacks of those which are based on the indications for performing a CS with categories that are not mutually exclusive and with low reproducibility for some of the most common conditions that lead to CS, such as fetal distress or dystocia.

The flexibility of the classification allows for the creation of subdivisions in each group that can improve analyses of local clinical practices. These suggestions are a critical contribution of this systematic review, providing clinicians, other health professionals and researchers with additional ideas to tailor the classification to their needs. Subdivisions have been proposed in almost all of the 10 Robson groups but it is clear that group 5 (women with a previous CS) is the group that received the largest number of suggestions (see Table 1). The recommended modifications in group 5 fall into one of two major axis: either the previous obstetric history of the woman (previous vaginal delivery or number of CS) or the onset of labour (spontaneous or other). In the current context of increasing numbers of caesarean deliveries, the contribution of the group of women with a previous CS (Group 5) to the overall rate of CS is critical from a clinical and epidemiological perspective to interpret practices and monitor the effectiveness of interventions. In addition, if users feel that more in depth analysis are needed, they can add the indications for CS, epidemiological information (e.g. BMI, age) and outcome (e.g. morbidity and mortality) within the 10 groups.

Despite its strengths, the Robson classification, users warn that it is not free of challenges and difficulties. The quality of the data and, therefore, the real value of using the classification should not be taken for granted as it is a struggle even in developed countries. Lack of definition or consensus on the core variables is an issue raised by several users. For example, it is necessary to reach an agreement on when labour starts and how to operationalize the difference between augmentation and induction of labor. Misclassification of women is a real threat and users recommend training, educational efforts and audits to avoid both misclassification and missing data. In fact, missing data has led some users to create a category “99” for these women. We believe this suggestion is very relevant and recommend the addition of this group to the Robson classification to make it completely “totally inclusive”. The size of this group “99” can be useful to audit the quality of the data.

The interpretation of the results of the classification is the weakest point of its use. A simple set of rules for interpretation was recently published by Robson [9] to help users explore all the information provided by this classification, especially when using it to compare data between different settings or changes over time. For example, it should be expected that the combination of groups 1 and 2 represents 35–42% of the total women and a high CS rate in group 2 (more than 35%) suggests a high pre-labour CS rate. Similarly, the combination of groups 3 and 4 should usually account for 30–40% of all women while group 9 should represent 0.2–0.6% of the total women and the CS rate in this particular group is expected to be 100%. However, these rules have not been validated and may not be applicable in all circumstances. The next crucial step would be to assess maternal and fetal outcomes *vs* CS rates in each of the 10 groups to be able to establish an optimal range of CS rate for best outcomes.

Strengths of this review start by its uniqueness. This is the first systematic review that analyses the experience of users related to pros and cons including challenges and recommendations. We developed a broad search strategy, in order to capture the largest possible number of publications on this topic and contacted the author of the classification to obtain unpublished material. We tried to reduce bias by extracting data in duplicate using a structured data-extraction form specifically created for this review, and by performing in triplicate the coding of the concepts, and the development of descriptive and analytical themes.

This systematic review has several limitations. Despite the efforts mentioned above, it is possible that we did not capture the full extent of its use since we are aware of users who are not documenting their experiences (Robson 2013, personal communication). We acknowledge that by trying to summarize studies and points of view from different settings and countries, the findings can be de-contextualized and what is applicable in one setting may not be relevant in others. However, we believe that most of the encountered barriers and proposed improvements would translate well into all contexts. In addition, despite the use of strict methodology at all steps of the systematic review, there is always potential for subjectivity in qualitative reviews of this type.

In the current international scenario of increasing rates of CS, the main drivers of this trend are still unclear and controversial. We believe that a CS rate can only be considered appropriate if the information is available to explain and justify it, and in this context, this systematic review provides important information, guidance and suggestions on how to use the Robson classification such as adding subdivisions and defining a new group for women with missing variables. By collecting real and timely data about which specific groups of women are having a CS, this classification can contribute to a better understanding of the drivers of increasing CS rates and to the development of effective interventions to safely curb this trend.

## Supporting Information

Checklist S1 **PRISMA Checklist.**
(DOC)

File S1 **Search strategy for electronic databases.**
(DOCX)

File S2 **Detailed description of the process to extract the concepts, create the themes and the final result.** In addition, for each Robson group, the detailed description of the sub-classifications proposed by authors.
(DOCX)
