# Supplementary material for: A Systematic Review of the Robson Classification for Caesarean Section: What Works, Doesn't Work and How to Improve It
Source: PLoS One. 2014 Jun 3;9(6):e97769. doi: 10.1371/journal.pone.0097769 (PMC4043665; doi:10.1371/journal.pone.0097769)
Supplement: File S1 — Search strategy for electronic databases. (DOCX) [file pone.0097769.s002.docx]

**File S1: Robson classification Search strategy**

Ran 1^st^ on Jan 18 2013.

PUBMED

#1 Robson*[All Fields] OR Ten-Group[All Fields] OR (Robson* classification*) OR (Ten stratified group*) OR (TGCS) OR (RTGCS) OR (the Robson Ten Group Classification System) OR (Robson Ten Group Classification) OR (Robson´s ten group classification) OR (Robson´s classification) OR (Robson cesarean delivery classification system) OR (Robson´s ten group classification (TGCS)) OR (Robson Ten Group Classification) OR (10-group) OR (10-group Robson Classification) OR (Robson Classes) OR (Ten Group Classification) = **10081**

#2 "Cesarean Section"[Mesh] OR (Cesarean Section*) OR (Cesarean Section rate OR (C-section) OR ((C-section) RATE) OR (Caesarean section rate*) OR (classifying caesarean section*) OR (Cesarean delivery rates) OR (Cesarean delivery) OR (Caesarean sections birth) OR (Delivery, Abdominal) OR (Abdominal Deliveries) OR (Deliveries, Abdominal) OR (Caesarean Section) OR (Caesarean Sections) OR (Abdominal Delivery) OR (C-Section (OB)) OR (C Section (OB)) OR (C-Sections (OB)) OR (Postcesarean Section) = **52078**

#1 AND #2 = 100

EMBASE

#1 Robson*[All Fields] OR Ten-Group[All Fields] OR (Robson* classification*) OR (Ten stratified group*) OR (TGCS) OR (RTGCS) OR (the Robson Ten Group Classification System) OR (Robson Ten Group Classification) OR (Robson´s ten group classification) OR (Robson´s classification) OR (Robson cesarean delivery classification system) OR (Robson´s ten group classification (TGCS)) OR (Robson Ten Group Classification) OR (10-group) OR (10-group Robson Classification) OR (Robson Classes) OR (Ten Group Classification) = **10081**

#2 "Cesarean Section"[Mesh] OR (Cesarean Section*) OR (Cesarean Section rate OR (C-section) OR ((C-section) RATE) OR (Caesarean section rate*) OR (classifying caesarean section*) OR (Cesarean delivery rates) OR (Cesarean delivery) OR (Caesarean sections birth) OR (Delivery, Abdominal) OR (Abdominal Deliveries) OR (Deliveries, Abdominal) OR (Caesarean Section) OR (Caesarean Sections) OR (Abdominal Delivery) OR (C-Section (OB)) OR (C Section (OB)) OR (C-Sections (OB)) OR (Postcesarean Section) =

**LILACS**

(Robson$) OR (Ten-Group) OR (Robson$ classification$) OR (Classificação de Robson) OR (Ten stratified group$) OR (TGCS) OR (RTGCS) OR (the Robson Ten Group Classification System) OR (Robson Ten Group Classification) OR (Robson´s ten group classification) OR (Robson´s classification) OR (Robson cesarean delivery classification system) OR (Robson´s ten group classification (TGCS)) OR (Robson Ten Group Classification) OR (10-group) OR (10-group Robson Classification) OR (Robson Classes) OR (Ten Group Classification) AND ((MH: **Cesárea) OR (Cesárea) OR (PARTO ABDOMINAL) OR (CESAREAN SECTION) OR (MH:**[E04.520.252.500](javascript:void(submit_GET_METHOD('002625','002625-1','hierarchic')))$))

_______________________________________________________________________

CINHAL Robson* OR Ten Group OR (Robson* classification*)

#1 Robson* OR Ten Group OR (Robson* classification*) OR (Ten stratified group*) OR (TGCS) OR (RTGCS) OR (the Robson Ten Group Classification System) OR (Robson Ten Group Classification) OR (Robson s ten group classification) OR (Robson s classification) OR (Robson cesarean delivery classification system) OR (Robson s ten group classification (TGCS)) OR (Robson Ten Group Classification) OR (10 group) OR (10 group Robson Classification) OR (Robson Classes) OR (Ten Group Classification)

#2 Cesarean Section OR (Cesarean Section*) OR (Cesarean Section rate OR (C section) OR ((C section) RATE) OR (Caesarean section rate*) OR (classifying caesarean section*) OR (Cesarean delivery rates) OR (Cesarean delivery) OR (Caesarean sections birth) OR (Delivery Abdominal) OR (Abdominal Deliveries) OR (Deliveries Abdominal) OR (Caesarean Section) OR (Caesarean Sections) OR (Abdominal Delivery) OR (C Section (OB)) OR (C Section (OB)) OR (C Sections (OB)) OR (Postcesarean Section)

#1 AND #2
