# Supplementary material for: A Systematic Review of the Robson Classification for Caesarean Section: What Works, Doesn't Work and How to Improve It
Source: PLoS One. 2014 Jun 3;9(6):e97769. doi: 10.1371/journal.pone.0097769 (PMC4043665; doi:10.1371/journal.pone.0097769)
Supplement: File S2 — Detailed description of the process to extract the concepts, create the themes and the final result. In addition, for each Robson group, the detailed description of the sub-classifications proposed by authors. (DOCX) [file pone.0097769.s003.docx]

**File S2**

**The detailed description of the process to extract the concepts, create the themes and the final result is depicted below.**

We have explained the process very succinctly in the methods section. We took, as a concept, any sentence in the original article that reflects or represents some form of judgement, opinion, view, belief or assessment (positive, negative or neutral), as well as recommendations and suggestions to improve the classification. Because of the particular nature of our research question, the concepts that we aimed to extract are not necessarily the findings of the original articles and useful concepts for this systematic review can be found anywhere in the original manuscripts (not just in the results or discussion).

The extracted concepts from each of the 73 articles were listed independently and, through discussions between three people, we arrived at a final proposal of three descriptive themes or topics in which the concepts extracted could be organized: 1) design and purpose of the classification; 2) Implementation of the classification; and 3) Interpretation of the information and data streaming from the implementation of the classification. These three descriptive themes formed the skeleton of the structure of the analysis and results.

The concepts extracted were then classified in one of each of the three descriptive themes where they best fitted. For each theme, the original concepts were combined to gather the same “notion, idea or thought” under the same “concept” and at the same time, be able to quantify the concept by counting the number of articles which capture the concept. After the reviewer’s suggestion to use the qualitative metasummary method, we were able to calculate the effect size of each concept by dividing the number of reports containing the concept (minus any report derived from the same study and therefore representing a duplicate) by the total number of reports (minus any report derived from the same study and therefore representing a duplicate). In our review, however, there were no duplicate reports.

The investigators conducted this process manually, i.e. without the use of specific software involving a large number of discussions before reaching full agreement. Three investigators conducted the process specified above with regular discussions and meetings until reaching full agreement.

**For each Robson group, the detailed description of the sub-classification proposed by authors follows:**

Subgroups were used or proposed by 28 studies [[1-28](#_ENREF_1)] and all but one subdivided Groups 2 and 4 into induced and CS before labor. Seven studies divided Group 5 into spontaneous labor, induced and CS before labor [[4](#_ENREF_4),[5](#_ENREF_5),[12](#_ENREF_12),[19](#_ENREF_19),[21](#_ENREF_21),[22](#_ENREF_22),[28](#_ENREF_28)]; six studies into women with one previous scar and women with more than one scar [[2](#_ENREF_2),[6](#_ENREF_6),[13](#_ENREF_13),[21](#_ENREF_21),[24](#_ENREF_24),[28](#_ENREF_28)]; three studies into women who have had a previous vaginal birth and those who have not [[21](#_ENREF_21),[24](#_ENREF_24),[28](#_ENREF_28)]; and one study proposed subdividing into women who attempted vaginal birth after CS (trial of labour after CS – TOLAC) and those who did not attempt labour. One study divided Group 6 into spontaneous labor, induced and CS before labor [[19](#_ENREF_19)]. Two divided group 7 into women with and without a previous scar [[6](#_ENREF_6),[13](#_ENREF_13)], and one into spontaneous labor, induced and CS before labor [[19](#_ENREF_19)]. Group 8 was divided into spontaneous labor, induced and CS before labor by four studies [[12](#_ENREF_12),[19](#_ENREF_19),[21](#_ENREF_21),[22](#_ENREF_22)], and into women with and without a previous scar by three [[6](#_ENREF_6),[13](#_ENREF_13),[21](#_ENREF_21)]. Two studies divided Group 9 into women with and without a previous scar [[6](#_ENREF_6),[13](#_ENREF_13)] and two into spontaneous labor, induced and CS before labor [[19](#_ENREF_19),[21](#_ENREF_21)]. Group 10 was subdivided by three studies into women with and without a previous scar [[6](#_ENREF_6),[13](#_ENREF_13),[21](#_ENREF_21)] and by three other studies into spontaneous labor, induced and CS before labor [[19](#_ENREF_19),[21](#_ENREF_21),[22](#_ENREF_22)]. One study divided group 1 into augmentation vs. no augmentation [[17](#_ENREF_17)] and in Canada, three studies added a so-called “group 99” for women who could not be classified because of missing or incomplete data [[4](#_ENREF_4),[23](#_ENREF_23),[29](#_ENREF_29)] and one added a “group 11” for singletons with unknown presentation [[5](#_ENREF_5)].

**Figure S1:** The Figure below depicts the different subgroups that were used or recommended by authors.


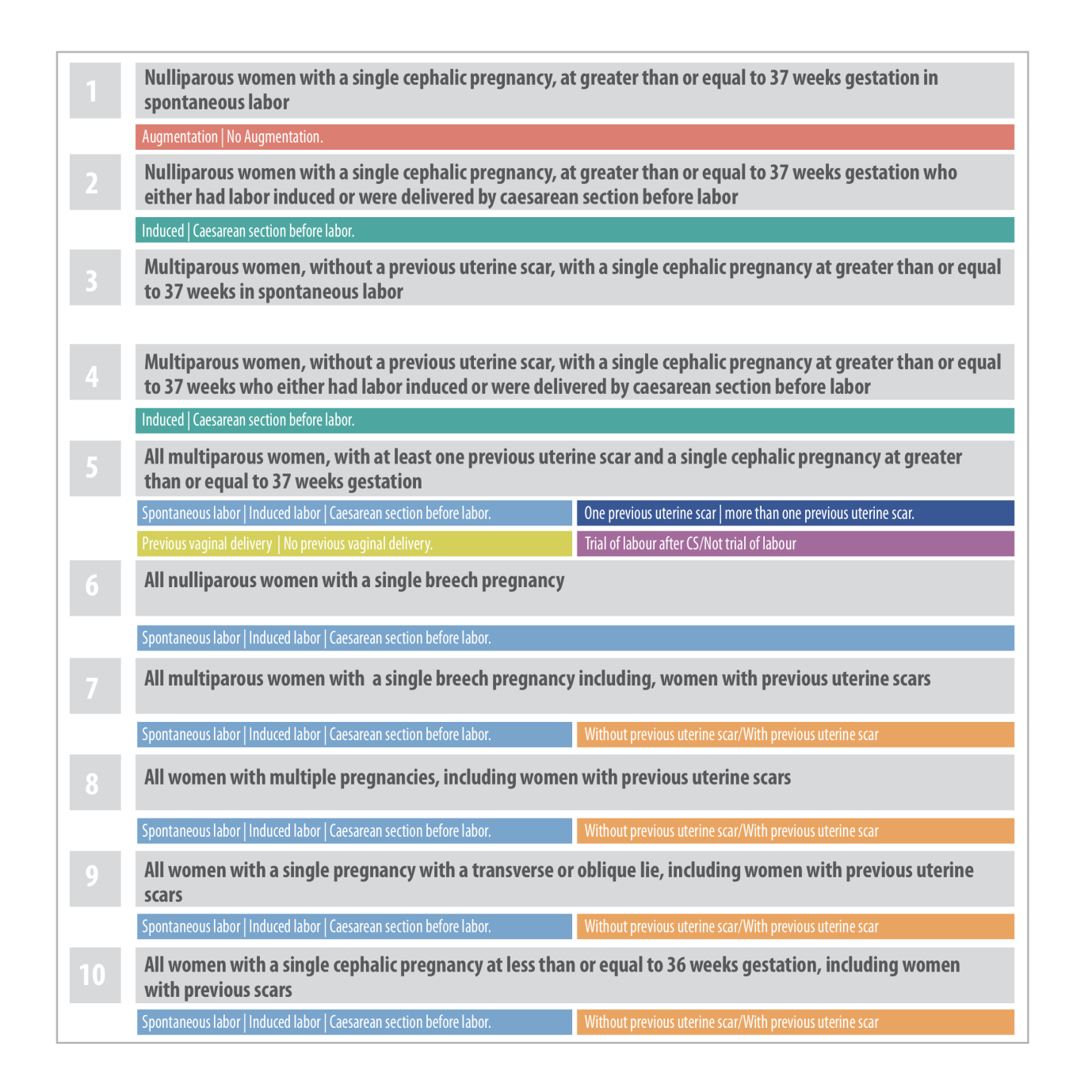


**References**

1. Brennan DJ, Murphy M, Robson MS, O'Herlihy C (2011) The Singleton, Cephalic, Nulliparous Woman After 36 Weeks of Gestation. Obstet Gynecol 117: 273-279.

2. Scarella A, Chamy V, Sepulveda M, Belizan JM (2011) Medical audit using the Ten Group Classification System and its impact on the cesarean section rate. Eur J Obstet Gynecol Reprod Biol 154: 136-140.

3. Zhang J, Troendle J, Reddy UM, Laughon SK, Branch DW, et al. (2010) Contemporary cesarean delivery practice in the United States. Am J Obstet Gynecol 203: e1-e10.

4. Fell D, Prince M, Sprague A, Walker M, Darling L, et al. (2011) Better outcomes registry and network (BORN) Ontario Perinatal Health Report 2009-2011, Greater Toronto Area LHINs 5 to 9.

5. Program BCPH (2009) Caesarean Birth Task Force Report 2008. Vancouver, BC, Canada.

6. Goonewardene M, Kumara DMA, Arachchi DRJ, Vithanage R, Wijeweera R (2012) The rising trend in caesarean section rates: should we and can we reduce it? Sri Lanka Journal of Obstetrics and Gynaecology 34: 11-18.

7. Bjarnadottir R, Smarason A (2013) Iceland Report from Birth Registry.

8. Gonzalez R, Merialdi M, Lincetto O, Lauer J, Becerra C, et al. (2006) Reduction in neonatal mortality in Chile between 1990 and 2000. Pediatrics 117: e949-e954.

9. Robson MS (2012) National Maternity Hospital Dublin. Clinical Report for the Year 2008.

10. Services AH (2009) Caesarean Births In Alberta. Alberta Perinatal Health Provincial Report. pp. 12-24.

11. Allen VM, Baskett TF, O'Connell CM (2010) Contribution of select maternal groups to temporal trends in rates of caesarean section. J Obstet Gynaecol Can 32: 633-641.

12. Slavin V, Fenwick J (2012) Use of a Classification Tool to Determine Groups of Women That Contribute to the Cesarean Section Rate: Establishing a Baseline for Clinical Decision Making and Quality Improvement. International Journal of Childbirth 2: 85-94.

13. Vera C, Correa R, Neira J, Rioseco A, Poblete A (2004) Utilidad de la evaluaci¢n de 10 grupos cl¡nicos obst‚tricos para la reducci¢n de la tasa de ces rea en un hospital docente. Revista Chilena de Obstetricia y Ginecología 69: 219-226.

14. Maneschi F, Sarno M, Vicaro V, Pane C, Ceccacci I, et al. (2009) Analisi Della Frequenza Di Taglio Cesareo Secondo Le Classi Di Rischio Clinico. Riv It Ost Gin 21: 13-18.

15. Thomas J, Paranjothy S (2001) Royal College of Obstetricians and Gynaecologists Clinical Effectiveness Support Unit. The National Sentinel Caesarean Section Audit Report. London, United Kingdom: RCOG Press.

16. Chan JCY, Honest H (2010) Implementing the ten-group-classification-system of ceasarean section at Good Hope Hospital (UK) for 2008. Arch Dis Child Fetal Neonatal.

17. Budhwa T, Holmberg V, Chapman B (2010) The Birthing Review Project.

18. Maneschi F, Sarno M, La Rocca A, Ceccacci I, Algieri M, et al. (2011) Riflessioni sul tasso globale di taglio cesareo. Epidemiologia NOG. pp. 4-9.

19. Farine D, Shepherd D (2012) Classification of caesarean sections in Canada: the modified robson criteria. J Obstet Gynaecol Can 34: 976-979.

20. Robson M, Hartigan L, Murphy M (2013) Methods of achieving and maintaining an appropriate caesarean section rate. Best Pract Res Clin Obstet Gynaecol 27: 297-308.

21. Robson MS (2001) Can we reduce the caesarean section rate? Best Pract Res Clin Obstet Gynaecol 15: 179-194.

22. Robson MS (2001) Classification of caesarean sections. Fetal and Maternal Medicine Review 12: 23-39.

23. Kelly S, Sprague A, Fell D, Murphy P, Aelicks N, et al. (2013) Examining Caesarean Section Rates in Canada Using the Robson Classification System. J Obstet Gynaecol Can.

24. Betran AP, Gulmezoglu AM, Robson M, Merialdi M, Souza JP, et al. (2009) WHO global survey on maternal and perinatal health in Latin America: classifying caesarean sections. Reprod Health 6: 18.

25. Howell S, Johnston T, Macleod SL (2009) Trends and determinants of caesarean sections births in Queensland, 1997-2006. Australian and New Zealand Journal of Obstetrics and Gynaecology 49: 606-611.

26. McCarthy FP, Rigg L, Cady L, Cullinane F (2007) A new way of looking at Caesarean section births. Aust N Z J Obstet Gynaecol 47: 316-320.

27. Sudarsan S, Soma S, Rupkamal D, Mayoukh C, Sekhar BH, et al. (2012) A Paradigm Shift to Check the Increasing Trend of Cesarean Delivery is the Need of Hour: But How? The Journal of Obstetrics and Gynecology of India 62: 391-397.

28. Costa ML, Cecatti JG, Souza JP, Milanez HM, Gulmezoglu MA (2010) Using a Caesarean Section Classification System based on characteristics of the population as a way of monitoring obstetric practice. Reprod Health 7: 13.

29. BC PS (2011) Examining cesarean delivery rates in British Columbia using the Robson Ten Classification. Part 1. Understanding the 10 groups. Vancouver, BC.
